# Mechanism of Resistance to Pyroxsulam in Multiple-Resistant *Alopecurus myosuroides* from China

**DOI:** 10.3390/plants11131645

**Published:** 2022-06-22

**Authors:** Yuning Lan, Ying Sun, Zhen Liu, Shouhui Wei, Hongjuan Huang, Yi Cao, Wenyu Li, Zhaofeng Huang

**Affiliations:** 1 State Key Laboratory for Biology of Plant Diseases and Insect Pests, Institute of Plant Protection, Chinese Academy of Agricultural Sciences, Beijing 100193, China; lan_yuning@163.com (Y.L.); suny264@163.com (Y.S.); m1587661417@163.com (Z.L.); weishouhui@caas.cn (S.W.); hjhuang@ippcaas.cn (H.H.); caoyi2622@163.com (Y.C.); lifreedomwy@163.com (W.L.); 2 College of Agriculture, Northeast Agricultural University, Harbin 150030, China

**Keywords:** *Alopecurus myosuroides*, pyroxsulam, ALS, mutation, target-site resistance, metabolic resistance

## Abstract

Black grass (*Alopecurus myosuroides* Huds.) is a highly competitive weed in winter wheat fields of China. Due to repeated use of acetolactate synthase (ALS) inhibitors, many *A. myosuroides* populations have evolved resistance to pyroxsulam in some wheat fields. Research was conducted to determine the molecular basis of herbicide resistance in the AH93 *A. myosuroides* population. Whole-plant dose–response assay confirmed that the AH93 population was resistant to pyroxsulam with a resistance index of 4.2. Cross- and multiple-resistance assays indicated that the AH93 population was cross-resistant to mesosulfuron-methyl and multiple-resistant to pinoxaden. Sequencing of the *ALS* and *ACCase* gene revealed that there was no target-site mutation in ALS, but Trp-2027-Cys and Cys-2088-Arg amino acid mutations in ACCase in the AH93 population. A malathion pretreatment study indicated that the AH93 population might have cytochrome P450–mediated herbicide metabolic resistance. This is the first report of pyroxsulam resistance in a multiple-resistant *A. myosuroides* population in China, and the Cys-2088-Arg mutation is the first reported case of an ACCase mutant conferring herbicide resistance in *A. myosuroides*.

## 1. Introduction

The increasing number of herbicide resistance cases in weeds is a major concern for sustainable agriculture development. To control these weeds, various herbicides with different sites of action have been applied to wheat fields. Over the last decade, the acetolactate synthase (ALS)-inhibiting herbicide pyroxsulam has been extensively used to manage grass weeds such as black grass (*Alopecurus myosuroides* Huds.) in wheat fields. However, repeated and intensive application of ALS-inhibiting herbicides poses great pressure for herbicide resistance selection in weeds. Herbicide resistance has made weed management more difficult and expensive. Therefore, clarifying the mechanism of herbicide resistance is helpful to maintain herbicide efficacy by formulating integrated resistance management strategies [1].

The mechanism of herbicide resistance includes target-site-based resistance (TSR) and nontarget-site-based resistance (NTSR) mechanisms. TSR is the result of target-site mutation or overexpression of herbicide target genes [2]. ALS-inhibiting herbicides are prone to be selected for resistance in weeds [3]. To date, 166 weed species have been reported to be resistant to ALS herbicides [4], and most of the reported cases display TSR, which is conferred by mutations in one of eight amino acid positions in ALS (Ala-122, Pro-197, Ala-205, Asp-376, Arg-377, Trp-574, Ser-653, and Gly-654) [5,6].

The NTSR mechanism includes reducing herbicide absorption and translocation in the plant or enhancing the degradation of herbicides through plant metabolism [7,8]. Many enzymes are associated with NTSR, of which cytochrome P450 monooxygenases are predominant and play a critical role in herbicide metabolic resistance [9,10]. P450s catalyse the detoxification of toxic xenobiotics such as herbicides, and detoxify and solubilize herbicide by modifying them with oxygen. Malathion, a cytochrome P450 inhibitor, has often been used as an indicator for herbicide metabolism by cytochrome P450s [11,12]. In some cases, NTSR may be present in weed populations that already contain TSR alleles [13,14].

In recent years, black grass has been frequently found in the Huang huai winter wheat growing areas, including the Chinese provinces of Hebei, Shandong, Anhui, and Henan, where it causes large yield reductions [15]. Recently, a putative resistant *A. myosuroides* population could not be managed by the field-recommended rates of pyroxsulam application in the winter wheat fields in Anhui Province, China. The current study aimed to determine the pyroxsulam resistance level and investigate the resistance basis to pyroxsulam in the *A. myosuroides* population.

## 2. Results

### 2.1. Whole-Plant Dose–Response Assays

**Figure 1:**
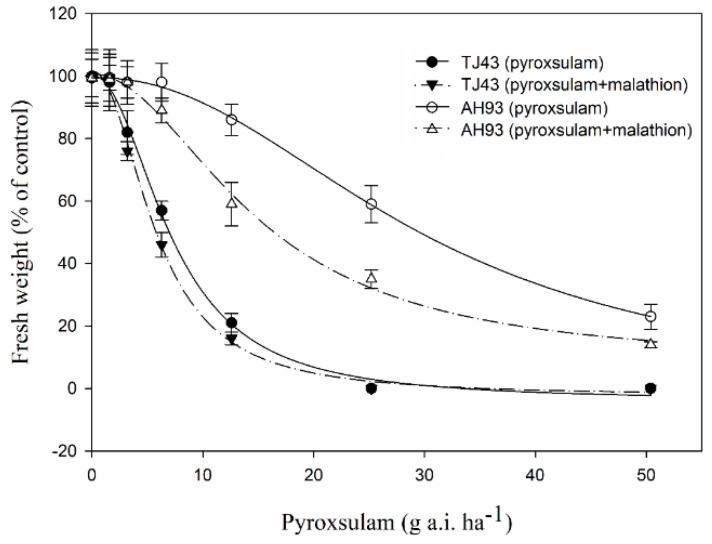
Dose–response curves after pyroxsulam treatment with or without malathion in the resistant (AH93) and susceptible (TJ43) black grass populations. Fresh weights were measured after 21 days of treatment and are represented as a percentage of the untreated control value.

The whole-plant dose responses of the susceptible TJ43 and resistant AH93 populations to pyroxsulam are represented in Figure 1. The results showed that the AH93 plants were not completely controlled by a pyroxsulam rate of up to 50.4 g ai ha^−1^, which was 4 times the recommended field rate. The GR_50_-based resistance level of the AH93 population was 4.2-fold higher than that of the TJ43 population (Table 1, Figure 1).

### 2.2. Effect of Malathion Pretreatment on Pyroxsulam Resistance

**Figure 2:**
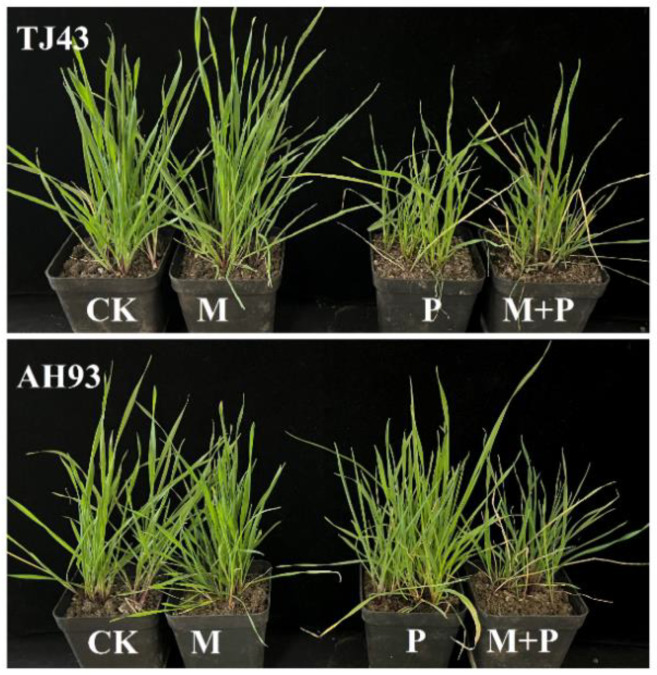
The resistant (AH93) and susceptible (TJ43) black grass plants 21 days after pyroxsulam treatment with or without malathion. CK, untreated; M, malathion (1000 g ai ha^−1^) treatment; P, pyroxsulam (12.6 g ai ha^−1^) treatment; M + P, malathion (1000 g ai ha^−1^) plus pyroxsulam (12.6 g ai ha^−1^) treatment.

When malathion was applied alone at 1000 g ai ha^−1^, no significant influence was found on seedling survival or biomass in either the TJ43 or AH93 plants. When malathion plus pyroxsulam was applied, the sensitivity to pyroxsulam in the AH93 plants greatly increased (Figure 1), and the GR_50_ value was reduced by 51% (Table 1). However, there was little difference between the effects on biomass of the TJ43 population after treatment with pyroxsulam (Figure 2).

### 2.3. Sensitivity to Other Herbicides

Herbicide screening was conducted to investigate the cross- and multiple-resistance patterns of the AH93 population. The results showed that all plants of the AH93 population survived mesosulfuron-methyl and pinoxaden when treated at the 1-, 2- and 4-fold recommended field doses. In contrast, all plants of the TJ43 population were killed at the three tested doses. Therefore, the AH93 population exhibited cross-resistance to mesosulfuron-methyl and multiple resistance to pinoxaden. In addition, the AH93 and TJ43 populations showed similar responses to cypyrafluone, and plants of both populations were 100% killed by cypyrafluone at the 2- and 4-fold field rates (Table 2).

### 2.4. ALS and ACCase Gene Sequencing

The *ALS* gene of *A. myosuroides* covering all eight known mutations was sequenced following PCR amplification. Analysis of the ALS sequences of ten individual AH93 plants revealed no nucleotide changes, resulting in amino acid substitutions. Thus, the known mutations in the *ALS* gene conferring target-site resistance to ALS inhibitors were not present in the AH93 population.

**Figure 3:**
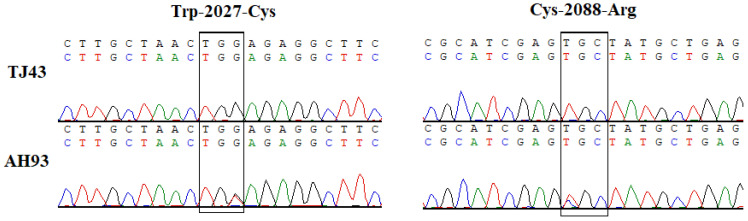
Partial *ACCase* gene comparison from the resistant (AH93) and susceptible (TJ43) black grass populations.

As the AH93 population showed multiple resistance to pinoxaden, the target *ACCase* gene was amplified and sequenced. Comparation of *ACCase* revealed a TGG to TGT substitution at amino acid position 2027 (Trp-2027-Cys) in eight individuals of the AH93 population. In addition, a TGC to CGC substitution at amino acid position 2088 (Cys-2088-Arg) was found in two individuals of the AH93 population (Figure 3). Both Trp-2027-Cys and Cys-2088-Arg mutation have double peaks and are heterozygous mutations.

## 3. Discussion

There have been some cases of resistance to ALS inhibitors among *A. myosuroides* in Europe and China. In previous studies, an altered ALS enzyme was reported as the main mechanism of resistance in these resistant populations [16]. Huang et al. reported that two *A. myosuroides* populations had evolved resistance to pyroxsulam with Pro-197-Thr or Trp-574-Leu mutation in ALS, but no resistance to ACCase herbicides [17]. This research confirms that an *A. myosuroides* population was cross-resistant to the ALS-inhibiting herbicides pyroxsulam and mesosulfuron-methyl. However, ALS sequencing analysis did not identify an amino acid mutation in ALS in this study. These results suggested that the resistance to pyroxsulam was likely not target-site-based. Therefore, we speculated that NTSR, such as enhanced metabolism, likely play a part in herbicide resistance.

Enhancing the degradation of herbicides by plant metabolism is another important herbicide resistance mechanism. It could confer unpredictable resistance to herbicides even with different modes of action. Cytochrome P450-mediated enhanced metabolism to ALS inhibitors has been documented in different weed species, such as *Lolium perenne* [18], *Beckmannia syzigachne* [19], and *Lolium rigidum* [20]. A previous study using green foxtail resistant to nicosulfuron revealed that nicosulfuron resistance could be reversed by malathion, an inhibitor of cytochrome P450s [7]. In this study, the resistance level to pyroxsulam was reduced by 51% by malathion (Table 1); this result suggested that herbicide metabolism mediated by cytochrome P450s might be involved in pyroxsulam resistance. However, it will be necessary to determine candidate cytochrome P450 genes associated with metabolic resistance to pyroxsulam in the AH93 population.

Different *A. myosuroides* populations resistant to ACCase-inhibiting herbicides have mainly been found in European countries [4]. To date, five different site mutations, Ile-1781-Leu, Trp-2027-Cys, Ile-2041-Asp, Asp-2078-Gly, and Gly-2096-Ala, have been reported in *A. myosuroides* resistant to ACCase inhibitors [17]. In this study, the AH93 *A. myosuroides* population could survive the pinoxaden application at 4× the field rate (240 g ha^−1^) and the Trp-2027-Cys and Arg-2088-Cys amino acid mutations were identified. The Trp-2027-Cys mutation has been reported in many grass weed species, such as *Digitaria insularis* [21] and *Rottboellia cochinchinensis* [22]. *A. myosuroides* with Trp-2027-Cys mutation from France could withstand pinoxaden application in the field rate (60 g ha^−1^) and showed cross-resistance to fenoxaprop and clodinafop [23]. In addition, The Cys-2088-Arg mutation is consistent with the findings of Papapanagiotou et al. [24], who reported that Cys-2088-Arg amino acid substitutions conferred resistance to ACCase herbicides in *Avena sterilis*. It is important to point out that this is the first report worldwide of the ACCase mutant Cys-2088-Arg that confers target-site-resistance in black grass. In this study, both Trp-2027-Cys and Cys-2088-Arg mutation have double peaks and are heterozygous mutations. Kaundun reported that herbicide-resistant levels depend on specific amino acid changes, the number of resistant alleles, and recommended field rates of herbicides [25]. Further study will isolate homogenous mutation subpopulations and investigate the resistance level between heterozygous and homozygous mutations via dose–response studies.

In recent years, resistant black grass populations have been established in the Huang huai winter wheat growing areas, including the Chinese provinces of Hebei, Shandong, Anhui, and Henan [15]. What is worse, the resistant AH93 population was multiple-resistant to ALS and ACCase herbicides. Many black grass populations resistant to ALS and ACCase herbicides due to target-site and non-target-site resistance mechanisms have been found, especially in Europe. Target-site mutations (Trp-2027-Cys and Cys-2088-Arg) and non-target-based resistance (enhanced metabolism) coexist in a single multiple resistant black grass population and was documented for the first time. In addition, through the result of sensitivity to other herbicides, the recommended rate of HPPD inhibitor cypyrafluone was not effective for the two black grass populations. This result indicated that black grass might be tolerant to cypyrafluone. However, it still needs to be verified by investigating more black grass populations. Therefore, this study is of great importance for farmers to devise resistance management strategies to stop or prevent the multiple-resistant black grass spreading. If the current weed management strategy does not change, especially by just increasing the herbicide dose, the AH93 population will cause an outbreak and spread to other fields via seed and pollen dispersal. This outcome will pose a serious threat to weed control and crop production. Therefore, over-reliance on a weed management system based solely on herbicides is not sustainable. In practice, the diversity of weed management, especially non-chemical strategies such as tillage practices, crop rotation, and cultural practices, is highly advocated.

## 4. Materials and Methods

### 4.1. Plant Material

Seeds from more than 100 individual plants of the putative pyroxsulam-resistant *A. myosuroides* population (AH93 33°04′ N, 117°04′ E) were collected in 2019 from a winter wheat field in Anhui Province, China. The wheat field had been treated with pyroxsulam during the year of seed collection. A known susceptible population (TJ43 39°46′ N, 117°13′ E) that had no history of herbicide application was used as a control. After air-drying, the seed samples were stored in glass bottles at room temperature.

Seeds were sown in 11 cm diameter plastic pots and grown in a greenhouse at 20 ± 3 °C for 12 h day and 15 ± 3 °C for a 12 h night with regular application of water and fertilizer. The seedlings were thinned to 8 plants per pot before herbicide treatment.

### 4.2. Whole-Plant Dose–Response Assays

When seedlings reached the 3–4-leaf stage, pyroxsulam was sprayed onto them using a laboratory sprayer equipped with a flat fan nozzle delivering 450 L ha^−1^. Pyroxsulam (4%; OD; Corteva; Shanghai, China) was applied at 0, 0.125×, 0.25×, 0.5×, 1×, 2×, and 4× for the susceptible TJ43 and resistant AH93 populations with × = 12.6 g ai ha^−1^. Three weeks after treatment, the aboveground plants were cut, and the biomass was measured. The experiment was repeated two times with three replications for each treatment.

### 4.3. Interaction between Pyroxsulam and Malathion

Seedlings of the TJ43 and AH93 populations at the 3–4-leaf stage were treated with malathion or malathion plus pyroxsulam. Malathion was applied at 1000 g ai ha^−1^ 1 h prior to pyroxsulam treatment. Pyroxsulam treatments followed the same methods and rates as those used in whole-plant dose–response assays. Untreated plants and plants treated with only malathion were used as controls. At 21 days after treatment (DAT), the aboveground biomass of the plants was determined. The experiment was repeated twice with three replications for each treatment.

### 4.4. Sensitivity to Other Herbicides

To estimate cross- and multiple-resistance patterns to other herbicides, seedlings of the TJ43 and AH93 populations at the 3–4-leaf stage were treated with the ALS inhibitor mesosulfuron-methyl (× = 13.5 g ai ha^−1^), the ACCase inhibitor pinoxaden (× = 60 g ai ha^−1^), or the HPPD inhibitor cypyrafluone (× = 180 g ai ha^−1^) at 1×, 2×, and 4× the recommended field dose (× corresponds to the recommended rate). Plant survival was recorded when plants had new growth or active tiller formation 21 days after treatment. Each herbicide treatment had four replicate pots (eight plants per pot), and the experiment was conducted twice.

### 4.5. Partial Sequencing of the ALS and ACCase Genes

Leaves from ten individuals of the TJ43 and AH93 *A. myosuroides* populations were harvested, and their DNA was extracted with a plant DNA kit (Tiangen Biotechnology Co., Ltd., Beijing, China). The *ALS* gene and *ACCase* gene were amplified according to the methods of Huang et al. [17] and Ge et al. [26].

The PCR products from the *ALS* gene and *ACCase* gene were confirmed on a 1% agarose gel, and then each PCR product was sequenced by the Beijing Genomics Institute (BGI, Beijing, China). Sequence data were aligned and compared using Vector NTI 12.5 (SigmaPlot Software Inc., Chicago, IL, USA).

### 4.6. Data Analysis

The data were expressed as a percentage of fresh weight compared to untreated control plants. Data were subjected to ANOVA and were pooled when no significant difference between the two experiments. Means of treatments were averaged and compared using Student’s *t*-test. Analysis of the dose–response data from the whole-plant assays were performed using SigmaPlot software (V12.0; SigmaPlot Software Inc., Chicago, IL, USA). The data were fitted to a nonlinear log-logistic model:

Y = C + [(D − C)/[1 + (X/GR_50_)^b^]

where C is the lower limit, D is the upper limit, and b is the slope at the GR_50_ (the herbicide dose required for 50% growth reduction). The level of resistance was determined by the resistance ratio, which was calculated as the GR_50_ of the resistant population divided by that of the susceptible population.

For cross- and multiple-resistance, the surviving rate was recorded as a percentage of survival plants compared to untreated control plants. And each herbicide treatment contained 32 replicate individuals.

## Figures and Tables

**Table 1:** GR_50_ values of pyroxsulam for the susceptible TJ43 and resistant AH93 *A. myosuroides* populations.

Treatment	TJ43 Population		AH93 Population		RI ^c^
	GR_50_ ^a^ (g ai ha^−1^) (SE) ^b^	*p*	GR_50_ (g ai ha^−1^) (SE)	*p*	
Pyroxsulam	7.22 (1.2)	0.0005	30.41 (2.5)	0.0012	4.2 a
Pyroxsulam + Malathion	6.83 (0.8)	0.0008	15.03 (1.9)	0.0026	2.2 b

^a^ GR_50_, herbicide concentration that caused a 50% reduction in plant growth. ^b^ SE, standard error. ^c^ RI, resistance index. Means followed by different letter in column are significantly different at *p* = 0.05.

**Table 2:** Plant survival rates of the TJ43 and AH93 *Alopecurus myosuroides* populations 21 days after herbicide treatment.

Population	Plant Survival Rate at 1×, 2× and 4× the Recommended Dose (%)								
	Mesosulfuron-Methyl (× = 13.5 g ai ha^−1^)			Pinoxaden (× = 60 g ai ha^−1^)			Cypyrafluone (× = 180 g ai ha^−1^)		
	1×	2×	4×	1×	2×	4×	1×	2×	4×
TJ43	0	0	0	0	0	0	40.6	0	0
AH93	100	100	100	100	100	100	43.8	0	0

## Data Availability

The data are available on request from the corresponding author.
